# Differences in Multi-Faceted Lifestyles in Response to the COVID-19 Pandemic and Their Association with Depression and Quality of Life of Older Adults in South Korea: A Cross-Sectional Study

**DOI:** 10.3390/nu13114124

**Published:** 2021-11-17

**Authors:** Kang-Hyun Park, Ah-Ram Kim, Min-Ah Yang, Ji-Hyuk Park

**Affiliations:** 1 Department of Occupational Therapy, College of Software and Digital Healthcare Convergence, Yonsei University, Wonju 26493, Korea; alcsro44@gmail.com; 2 Super-Aged Society New Normal Lifestyle Research Institute, Yonsei University, Wonju 26493, Korea; 3 Department of Occupational Therapy, Graduate School, Yonsei University, Wonju 26493, Korea; aramkim495@gmail.com; 4 Korea Health Industry Development Institute, Cheongju-si 28159, Korea; minamam6@gmail.com

**Keywords:** lifestyle, COVID-19 pandemic, depression, quality of life, older adults

## Abstract

Background: The World Health Organization declared COVID-19 a global pandemic on 11 March 2020, due to the number of newly reported confirmed cases and the rapid increase in deaths. Therefore, countries around the world limited their population to policies such as “social distancing” or “staying at home” to prevent the spread of the virus. The purpose of this study was to evaluate differences in lifestyle pre and post the outbreak COVID-19 among older adults in South Korea and to identify the impact of lifestyle differences on depression and quality of life. Methods: An online single questionnaire covering sociodemographic data, lifestyle details, depression status, and quality of life level was distributed using mailing lists and social media. To assess lifestyles differences in older people pre and post the outbreak of COVID-19 pandemic, the online single questionnaire was used post COVID-19 pandemic. Based on the participants’ memories, they responded lifestyles at two time points (pre and post COVID-19 pandemic). Results: The results showed that there was a significant decrease in physical activity and activity participation during the pandemic. In terms of nutrition, there was no statistically significant change pre and post the outbreak COVID-19, except for the intake of protein, fat, and vitamins. Additionally, the results showed that the resulting lifestyle differences seem to have had a negative impact on depression and quality of life among older adults in South Korea. Conclusion: There was a significant difference the lifestyle patterns among the participants in South Korea between the current period and pre COVID-19 pandemic. Additionally, it is observed that these differenced lifestyles were associated with depression and quality of life among the participants. Our findings may help to develop public health programs that support healthy lifestyles in pandemic conditions.

## 1. Introduction

The coronavirus disease 2019 (COVID-19) is an acute respiratory syndrome that emerged in Wuhan, China, in late 2019 [1]. On March 11, 2020, the World Health Organization (WHO) declared COVID-19 a global pandemic due to the number of newly reported confirmed cases and the rapid increase in deaths [2]. By the beginning of August 2021, more than 200 million COVID-19 cases were reported worldwide of which approximately 2.2 million were in South Korea [3,4]. Therefore, countries around the world started limiting their population to policies such as “social distancing” or “staying at home” to prevent the spread of the virus [4,5].

Social distancing or staying at home to prevent the spread of COVID-19 leads to isolation, which is likely to adversely affect individuals’ physical and mental health [6]. Likewise, the more time people spend at home, the more intense the resulting mental, emotional, and lifestyle problems [7]. Older adults are frequently unable to adapt to isolation measures and suffer from depression and cognitive complaints [8]. However, the health of older adults needs to be protected during the pandemic because no definite treatment for COVID-19, except for vaccination, has yet been developed. Therefore, it is vital to prioritize preventive approaches to maintain health and well-being [9].

From a prevention perspective, it is important to maintain a healthy lifestyle [10]. Lifestyle has been defined diversely and comprehensively in prior research. Lifestyle factors based on the WHO’s definition of terminology include drinking, smoking, exercise, nutrition, and stress [11]. According to Park et al. [12], lifestyle can be classified according to a person’s life patterns, and can be defined as a multi-faceted concept that involves a person’s consciousness of life, values, and character; appropriate lifestyle changes concerning physical activity, nutrition, activity participation, sleep, smoking, and alcohol intake may help shift the population distribution of infection risk and aid in preventing COVID-19 [13].

Social distancing or staying at home due to COVID-19-related isolation may lead to a variety of unhealthy lifestyles, such as the adoption of an unbalanced diet, a reduction in physical activity, increased use of alcohol and tobacco, and an increase in screen time, causing impaired sleep patterns [13]. All unhealthy lifestyles are related to non-communicable diseases and can interfere with immunity in older adults.

Therefore, the purpose of this study was to identify lifestyle differences in older people pre and post the outbreak of COVID-19 pandemic which were assessed the via a single questionnaire collected post COVID-19 pandemic. Additionally, we would like to investigate whether lifestyle differences pre and post COVID-19 pandemic affect people’s depression and quality of life. The results of this study will help support healthy lifestyles and assist in the development of effective intervention plans for individuals and countries.

## 2. Materials and Methods

### 2.1. Study Design and Participants

This study was designed as a cross-sectional survey. It was conducted online survey due to the restrictions imposed by social distancing introduced to prevent the spread of COVID-19. The survey was conducted by a specialized online research company (www.embrain.com, accessed on 17 November 2021). The company has 1,428,252 research panels, which are composed of respondents who have previously expressed their intention to participate in the survey and provided personal information through contracts with research company. Participants in the study were older adults aged 55 years and above in South Korea. The inclusion criteria for the study were (1) aged 55 years or older living in the community, (2) able to communicate fluently, (3) able to read Korean, and (4) voluntarily agreed to participate in the study. Individuals who agreed to participate checked the consent tick box on the first page of the survey form. Participants completed online questionnaires and answered questions about lifestyle differences using the Yonsei Lifestyle Profile (YLP) developed based on prior research [12]. The participants were required to answer their lifestyle including physical activity, activity participation and nutrition pre and post COVID-19 pandemic through the YLP questionnaire collected post COVID-19 pandemic. This study was conducted from 18 to 22 September 2020. It was approved by the Institutional Review Board of Yonsei University Mirae Campus (approval number: 1041849-202109-SB-147-02).

### 2.2. Questionnaire

#### 2.2.1. Sociodemographic Data

The sociodemographic data for the study were (1) gender (female, male), (2) age (years), (3) years of education (elementary school, middle degree, high school, and college or university), (4) retirement status (yes or no), (5) living alone (yes or no), and (6) chronic disease status (yes or no). The demographic characteristics of the study sample (*n* = 327) are presented in Table 1.

#### 2.2.2. Lifestyle

This study measured the multi-faceted lifestyle of older adults in Korea using the YLP questionnaire developed in a previous study [12]. The YLP included 60 questions covering: (1) physical activity, (2) participation in activities, and (3) nutrition. Participants responded to the frequency of participation in a specific activity and the number of times they ate a particular food in a week. The YLP reflected high internal reliability, with a Cronbach’s α of 0.83. The intraclass correlation coefficient was 0.97 for the total score of the YLP regarding test-retest reliability [12]. In this research, to examine the differences in terms of the participants’ lifestyle starting from the COVID-19 outbreak, The YLP questionnaire was modified (Supplementary File S1). The participants are required to recall their lifestyles pre and post COVID-19 pandemic respectively after COVID-19 pandemic. In other word, the participants self-reported their lifestyles of period pre and post COVID-19 pandemic via a single questionnaire (YLP) collected post COVID-19 pandemic. The internal reliability of the modified YLP in the present study was a Cronbach’s α of 0.803.

#### 2.2.3. Depression

The Korean version of the Center for Epidemiological Studies Scale (CES-D) was used to measure depression levels. The Korean version was developed by Chon and Rhee [14] and the original version is based on Radloff’s [15] depression scale developed by the National Institute of Mental Health. The questionnaire included 20 questions covering: (1) depressed affect, (2) positive affect, (3) somatic complaints, and (4) interpersonal problems. Each question consisted of a four-point Likert scale, and the higher the total score, the higher the depression level. The internal reliability of the CES-D was 0.89, and the test-retest reliability was 0.93 [14].

#### 2.2.4. Quality of Life

The Korean version of the World Health Organization Quality of Life-Life Brief (WHOQOL-BREF) was used to measure quality of life. The Korean version was developed by Min et al. [16] and is based on the Quality of Life Scale [17] developed by the WHO in 1998. The WHOQOL-BREF contains 26 questions covering four domains: physical health, psychological health, social relationships, and environmental health, as well as quality of life and general health items. The higher the total score, the higher the quality of life, with the Korean version of the WHOQOL-BREF producing a Cronbach’s α value of 0.898 [16].

### 2.3. Statistical Analysis

This study’s findings were analyzed using descriptive statistics and the data from the survey which were conducted after the COVID-19 pandemic were used. Because the survey was performed after COVID-19 pandemic, and participants were asked to respond based on their memories regarding lifestyles, depression, and quality of life during the pre and post COVID-19 pandemic respectively via a single questionnaire. A paired *t*-test was used to analyze the correlation. Odds ratios (ORs) and 95% confidence intervals (CIs) for frailty, depression, and quality of life were analyzed using logistic regression analysis. All *p*-values were two-sided, and a *p*-value < 0.05, was regarded as statistically significant. All statistical analyses were performed using SPSS software version 25.0 (SPSS Institute, Cary, NC, USA).

## 3. Results

### 3.1. Sociodemographic Data of the Study Participants

The general characteristics of the study samples are presented in Table 1. In total, 327 community-dwelling elderly (mean age 60.40 ± 4.60 years) participated in the survey. In terms of gender distribution, 50.9% were female and 49.2% were male. The majority of the participants had graduated from college or university (76.6%). Additionally, a high percentage of polled individuals lived with family members during the COVID-19 pandemic; only 7.6% lived alone.

### 3.2. Effect of the COVID-19 Pandemic on Physical Activity

**Figure 1:**
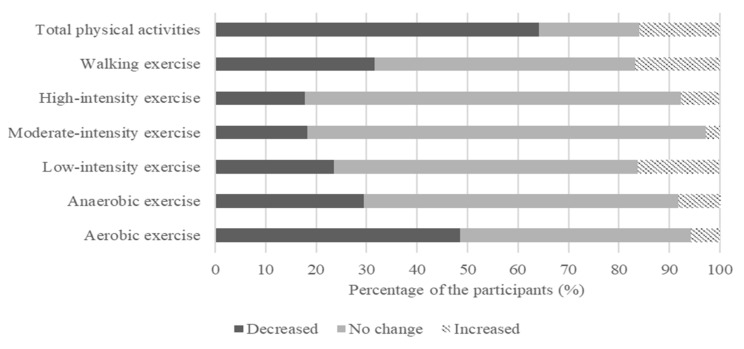
Percentage of population that reported differences in physical activity pre and post the outbreak of COVID-19.

The impact of the COVID-19 pandemic on physical activity was measured using the YLP questionnaire (Table 2). There was a significant decrease in all six exercise types, including aerobic (*p* < 0.001), anaerobic (*p* < 0.001), low-intensity (*p* < 0.01), moderate-intensity (*p* < 0.001), high-intensity (*p* < 0.01), and walking (*p* < 0.001). This shows a significant decline in physical activity during the COVID-19 pandemic when the population was required to stay at home and adhere to social distancing policies to prevent spreading infection: a decrease in the frequency of the six types of exercise during the pandemic was recorded by 64% of participants (Figure 1).

### 3.3. Effect of the outbreak of COVID-19 Pandemic on Activity Participation

**Figure 2:**
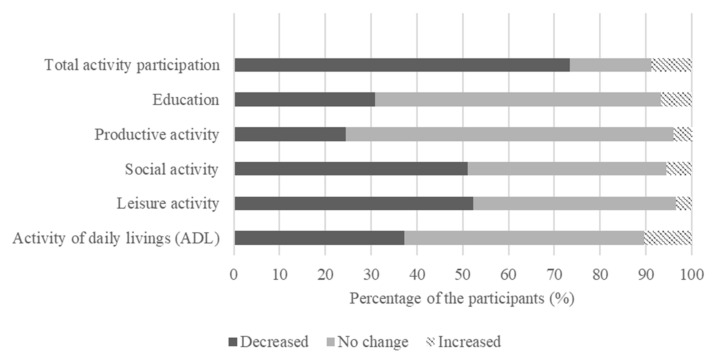
Percentage of population that reported differences in activity participation pre and post the outbreak of COVID-19.

Meaningful activity participation pre and post COVID-19 was also measured using the YLP questionnaire (Table 3). As a result, the number of days those respondents spent participating in activities of daily living (ADL) and leisure, social, productive, and educational pursuits decreased significantly (*p* < 0.001). When compared to activity pre the COVID-19 pandemic period, 73.4% of participants reported a decline in activity participation while only 8.9% of the participants showed an increase (Figure 2).

### 3.4. Effect of the COVID-19 Pandemic on Nutrition

**Figure 3:**
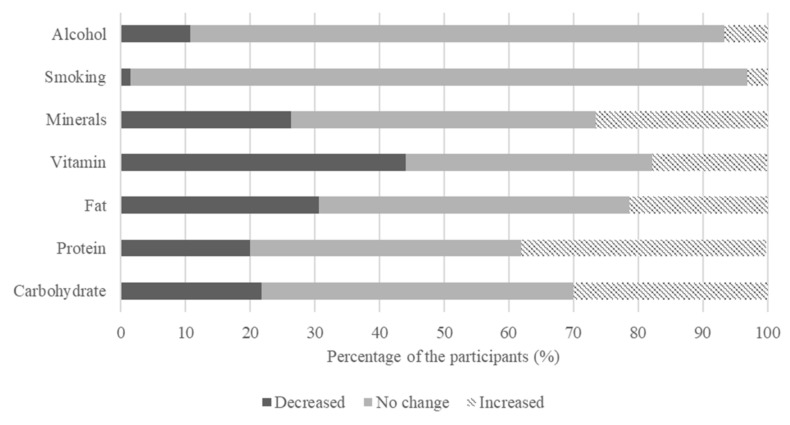
Percentage of population that reported differences in nutritional intake frequency pre and post the outbreak of COVID-19.

Concerning nutrition, there was no statistically significant change pre and post the outbreak of COVID-19, except for the intake of protein, fat, and vitamins (Table 4). It was observed that those who increased their intake of proteins such as chicken, tofu, beans, or eggs (*p* < 0.001), decreased their frequency of consumption of fats such as sesame oil, nuts, and butter (*p* < 0.05), and vitamins including green vegetables, fruits, and seaweed (*p* < 0.001). The majority of participants indicated no change in smoking (95.4%) and drinking habits (82.6%) pre and post the outbreak of COVID-19 (Figure 3).

### 3.5. Associations between Differences in Lifestyle and Psychological Health

Associations between dissimilarity in lifestyle and psychological health are shown in Table 5. Participants who showed a decline in physical activity during the COVID-19 pandemic were more likely to have a lower quality of life (adjusted OR = 1.473, 95% CI = 1.033–2.099). In terms of activity participation, those who reported a decrease were more likely to have a higher level of depression (adjusted OR = 0.960, 95% CI = 0.934–0.987) and lower quality of life (adjusted OR = 1.847, 95% CI = 1.247–2.737). Similarly, participants who showed a decrease in nutrition intake were more likely to have a higher level of depression (adjusted OR = 0.974, 95% CI = 0.953–0.996) and lower quality of life (adjusted OR = 1.684, 95% CI = 1.185–2.392).

## 4. Discussion

The characteristics pre and post the outbreak of COVID-19 in the study variables were as follows. First, for physical activity, the number of participation days in all detailed areas significantly decreased post the outbreak COVID-19. This was consistent with studies showing that national policies such as social distancing and isolation limit the physical activity of the elderly [18]. According to Rodríguez et al. [19], it is important to continue physical activity during isolation due to COVID-19, which supports the results of this study. Thus, a simple and safe physical activity program for the elderly, such as a home-based training program that is not affected by social distancing regulations is essential.

Second, the outbreak of COVID-19 resulted in the decline of activity participation. According to Pizarro-Pennaroli et al. [20], people spent more time at home, thereby reducing the performance of ADL by older people due to restrictions. In addition, this study identified that social distancing restricted leisure, social, educational, and productive activities, thereby reducing freedom and autonomy [21,22,23]. As social distancing restricts senior citizens from participating in activities, computer-based video programs are needed to allow them to experience various leisure and educational pursuits.

Third, there have been no significant differences in nutrition since the outbreak of COVID-19. Protein consumption, including chicken and eggs, has increased due to social distancing or isolation, which can be attributed to an increase in takeaway food orders [24]. In addition, the consumption of fats and vegetables, including sesame oil and nuts, and vitamin intake, have decreased since the outbreak of COVID-19; this can be attributed to the inability to go to the supermarket due to COVID-19 restrictions and dependence on food deliveries. Among lifestyle factors, nutrition is essential for healthy old age and is important in preventing and maintaining health [25]. Therefore, education on the importance of nutrition is needed in preparation for old age, as well as specific advice on nutrients considering individuals’ underlying diseases.

Finally, the results of this study showed that the decline in the number of days of physical activity and activity participation due to COVID-19 has led to higher levels of depression and lower quality of life. It also showed that the more unbalanced a person’s nutrition, the higher their level of depression and the lower their quality of life. Studies show that older people who regularly engage in physical activity are less likely to be exposed to depression [26] and that regular physical activity is essential for older people [27]. Older people participating in a variety of activities are less likely to suffer from depression, and those who have active lives show reduced levels of depression and improved quality of life [28,29]. In addition, good nutrition increases in importance with age and older people with balanced nutrition show lower levels of depression [30] and higher quality of life [31].

Through this study, we identified the impact of lifestyle on depression and quality of life in elderly people post the outbreak of COVID-19. Maintaining a healthy lifestyle is important in terms of prevention [32], and it is now increasingly important in the prevention of infectious diseases such as COVID-19. However, due to the development of a contact-free society, the elderly have difficulty managing their lifestyles compared to other age groups [33]. To address this, information and communication technology (ICT) needs to be utilized to address depression and quality of life improvements in older people. In addition, older people can have difficulty using computers or smartphones, thus training in computer use needs to be provided so that ICT-based activity and education programs can be utilized.

## 5. Limitations

This study had several limitations. First, it may not have provided an adequate comparison between the pre- and post-COVID-19 situation because the survey was conducted post the outbreak. Additionally, the lifestyle measurement called the YLP was modified to capture the differences in lifestyle of the participants. The Original version of the YLP was asked to the respondent’s lifestyle in the last week, but in this research, the participants required to recall the past few months for answering the lifestyle pre the starting COVID-19 pandemic. Because the human memory is highly vulnerable, it is evitable that the recall bias can occurs when the participants remember their lifestyles. However, at the start of the COVID-19 pandemic, the situation was changed rapidly depends on the nations. As a result, it was not possible to gather true pre-pandemic responses. Thus, the present results of the study should be interpreted carefully. Second, it is difficult to apply these findings to all elderly people due to the relatively low average age and high educational level of the respondents; only those who could participate in the online survey were eligible to take part, which may have excluded older people with limited ICT skills. Finally, controlling factors that affect depression and quality of life have not been accounted for in this study.

## 6. Conclusions

In conclusion, our data demonstrates that large-scale isolation strategies to control a pandemic resulting in low levels of physical activity, activity participation, and unhealthy diets among older adults in Korea are associated with depression and a lower level of quality of life. These results suggest that prolonged confinement due to COVID-19 promotes unhealthy lifestyle differences, which in the long term could have a negative impact on public mental health and quality of life. Thus, we suggest that public health programs that build and support a healthy and balanced lifestyle during future mass lockdowns should be developed. Additionally, ongoing evaluation and monitoring of the impact of lockdown rules and social distancing which is associated with the COVID-19 pandemic on healthy lifestyle patterns is required to develop health promotion strategies.

## Figures and Tables

**Table 1:** General characteristics of the study participants (*n* = 327).

Variables	*N*	Percentage or Mean (SD)
Gender		
Male	161	49.2
Female	166	50.8
Age	327	60.40(4.60)
Age group		
55–59	163	49.8
60–69	150	45.9
>70	14	4.3
Years of Education		
0 (no formal education)	1	0.3
1–6 (elementary)	1	0.3
7–9 (middle school)	7	2.2
10–12 (high school)	74	22.6
>13 (college or University)	244	76.6
Retirement status		
Yes	162	49.5
No	165	50.5
Living Alone		
Yes	25	7.6
No	302	92.4
Chronic disease status		
Yes	174	53.2
No	153	46.8

**Table 2:** Self-reported physical activity of periods pre and post COVID-19 pandemic which were assessed via a single questionnaire collected post COVID-19 pandemic (*n* = 327).

	Pre	Post	*t* (95% CI)
	Mean (SD)	Mean (SD)	
Aerobic exercise	2.84 (1.10)	2.17 (1.15)	11.60 (0.56–0.79) ***
Anaerobic exercise	2.08 (1.08)	1.77 (0.98)	5.59 (0.20–0.42) ***
Low-intensity exercise	2.96 (1.13)	2.83 (1.16)	2.62 (0.03–0.23) **
Moderate-intensity exercise	1.49 (0.82)	1.24 (0.53)	6.19 (0.17–0.32) ***
High-intensity exercise	1.66 (0.93)	1.54 (0.91)	3.07 (0.04–0.20) **
Walking exercise	3.23 (1.12)	2.99 (1.14)	4.16 (0.12–0.35) ***
Total physical activity	2.38 (0.63)	2.09 (0.58)	9.97 (0.23–0.34) ***

*** *p* < 0.001; ** *p* < 0.01.

**Table 3:** Self-reported activity participation of periods pre and post COVID-19 pandemic which were assessed via a single questionnaire collected post COVID-19 pandemic (*n* = 327).

	Pre	Post	*t* (95% CI)
	Mean (SD)	Mean (SD)	
Activities of daily living	3.52 (1.03)	3.09 (1.16)	7.52 (0.31–0.53) ***
Leisure activity	1.98 (0.85)	1.37 (0.72)	13.92 (0.53–0.69) ***
Social activity	2.03 (0.84)	1.47 (0.75)	11.90 (0.46–0.65) ***
Productive activity	2.99 (1.30)	2.63 (1.38)	7.23 (0.26–0.46) ***
Education	1.71 (0.81)	1.42 (0.76)	6.72 (0.20–0.37) ***
Total activity participation	2.44 (0.53)	2.00 (0.56)	15.58 (0.39–0.50) ***

*** *p* < 0.001.

**Table 4:** Self-reported nutritional intake frequency of periods pre and post COVID-19 pandemic which were assessed via a single questionnaire collected post COVID-19 pandemic (*n* = 327).

	Pre	Post	*t* (95% CI)
	Mean (SD)	Mean (SD)	
Carbohydrate	2.88 (0.55)	2.92 (0.58)	−1.82 (−0.09–0.00)
Protein	2.55 (0.68)	2.66 (0.66)	−4.45 (−0.15–0.06) ***
Fats	2.29 (0.66)	2.24 (0.68)	2.30 (0.01–0.89) *
Vitamins	3.24 (0.76)	3.06 (0.76)	6.56 (0.13–0.23) ***
Minerals	2.37 (0.78)	2.41 (0.78)	−1.51 (−0.80–0.01)
Total intake of five nutrients	2.67(0.50)	2.66(0.52)	0.657(−0.02–0.04)
Smoking	1.53 (1.27)	1.54 (1.28)	−0.60 (−0.05–0.03)
Alcohol	1.74 (0.91)	1.70 (0.91)	1.50 (−0.01–0.09)

*** *p* < 0.001; * *p* < 0.05.

**Table 5:** Associations between variation in lifestyle and psychological health.

	Depression	Quality of Life
	Logistic Regression-Odd Ratio (95% CI)	
Physical activity	0.977 (0.955–1.001)	1.473 (1.033–2.099) *
Activity participation	0.960 (0.934–0.987) **	1.847(1.247–2.737) **
Nutrition	0.974(0.953–0.996) *	1.684 (1.185–2.392) **

Reference for logistic regression is “no change/increased group.” * *p* < 0.05, ** *p* < 0.01.

## Data Availability

Data sharing is not applicable to this article due to ethical policy.

## Supplementary Materials

The following are available online at https://www.mdpi.com/article/10.3390/nu13114124/s1, Supplementary File S1. The Yonsei Lifestyle Profile (English version).
