# Supplementary material for: Differences in Multi-Faceted Lifestyles in Response to the COVID-19 Pandemic and Their Association with Depression and Quality of Life of Older Adults in South Korea: A Cross-Sectional Study
Source: Nutrients. 2021 Nov 17;13(11):4124. doi: 10.3390/nu13114124 (PMC8625190; doi:10.3390/nu13114124)
Supplement: Supplementary file 1 [file nutrients-13-04124-s001.zip › nutrients-1432128-supplementary.pdf]

Supplementary File S1. The Yonsei Lifestyle Profile (English version)

The Yonsei Lifestyle Profile

I. Physical activity

The following are questions about physical activity. Indicate how many times you participate in the following activities per week.

Please consider your engagement in these activities before and after the onset of the COVID-19 pandemic and mark (∨) your answer on the box next to the choice that is most applicable for you.

1. This is a question about aerobic exercise in the last week.

(※ Aerobic exercise: A relatively long, continuous breathing exercise, such as jogging, aerobics, jump rope, etc.)

1.1) (Before COVID-19) How many days did you engage in aerobic exercises in the last week?

☐ **Never** ☐ **1–2 days** ☐ **3–4 days** ☐ **5–6 days** ☐ **Every day**

1.2) (After COVID-19 onset) How many days did you engage in aerobic exercises in the last week?

☐ **Never** ☐ **1–2 days** ☐ **3–4 days** ☐ **5–6 days** ☐ **Every day**

2. This is a question about anaerobic exercise in the last week.

(※ Anaerobic exercise: Short-duration workouts, such as sprinting and push-ups, which leave one short of breath, and are difficult to sustain.)

2.1) (Before COVID-19) How many days did you engage in anaerobic exercises in the last week?

☐ **Never** ☐ **1–2 days** ☐ **3–4 days** ☐ **5–6 days** ☐ **Every day**

2.2) (After COVID-19 onset) How many days did you engage in anaerobic exercises in the last week?

☐ **Never** ☐ **1–2 days** ☐ **3–4 days** ☐ **5–6 days** ☐ **Every day**

3. This is a question about low-intensity physical activity in the last week.

(※ Low-intensity physical activity: physical activity equivalent to 2–2.9 MET, such as gardening, house-cleaning, fishing, etc.)

3.1) (Before COVID-19) How many days did you engage in low-intensity physical activities in the last week?

☐ **Never** ☐ **1–2 days** ☐ **3–4 days** ☐ **5–6 days** ☐ **Every day**

3.2) (After COVID-19 onset) How many days did you engage in low-intensity physical activities in the last week?

☐ **Never** ☐ **1–2 days** ☐ **3–4 days** ☐ **5–6 days** ☐ **Every day**

4. This is a question about <u>moderate-intensity physical activity</u> in the last week.

(※ Moderate-intensity physical activity: physical activity equivalent to 3–5.9 MET. such as swimming, doubles tennis, badminton, etc.)

4.1) (Before COVID-19) How many days did you engage in moderate-intensity physical activities in the last week?

☐ **Never** ☐ **1–2 days** ☐ **3–4 days** ☐ **5–6 days** ☐ **Every day**

4.2) (After COVID-19 onset) How many days did you engage in moderate-intensity physical activities in the last week?

☐ **Never** ☐ **1–2 days** ☐ **3–4 days** ☐ **5–6 days** ☐ **Every day**

5. This is a question about <u>high-intensity physical activity</u> in the last week.

(※ High-intensity physical activity: physical activity equivalent to 6–9.9 MET, such as running, climbing, cycling, etc.)

5.1) (Before COVID-19) How many days did you engage in high-intensity physical activities in the last week?

☐ **Never** ☐ **1–2 days** ☐ **3–4 days** ☐ **5–6 days** ☐ **Every day**

5.2) (After COVID-19 onset) How many days did you engage in high-intensity physical activities in the last week?

☐ **Never** ☐ **1–2 days** ☐ **3–4 days** ☐ **5–6 days** ☐ **Every day**

6. This is a question about going on walks in the last week.

6.1) (Before COVID-19) How many days did you spend walking in the last week?

☐ **Never** ☐ **1–2 days** ☐ **3–4 days** ☐ **5–6 days** ☐ **Every day**

6.2) (After COVID-19 onset) How many days did you spend walking in the last week?

☐ **Never** ☐ **1–2 days** ☐ **3–4 days** ☐ **5–6 days** ☐ **Every day**

II. Activity participation

The following are questions about your participation in certain activities. Indicate how often you have participated in the following physical activities in the last week.

Please consider your engagement in these activities before and after the onset of the COVID-19 pandemic, and mark (V) your answer on the box next to the choice that is most applicable for you.

1. This is a question about activities of daily living (ADLs) in the last week.

1.1) (Before COVID-19) How many days did you participate in ADLs in the last week?

☐ **Never** ☐ **1–2 days** ☐ **3–4 days** ☐ **5–6 days** ☐ **Every day**

1.2) (After COVID-19 onset) How many days did you participate in ADLs in the last week?

☐ **Never** ☐ **1–2 days** ☐ **3–4 days** ☐ **5–6 days** ☐ **Every day**

2. This is a question about leisure activity in the last week.

2.1) (Before COVID-19) How many days did you participate in leisure activities in the last week?

☐ **Never** ☐ **1–2 days** ☐ **3–4 days** ☐ **5–6 days** ☐ **Every day**

2.2) (After COVID-19 onset) How many days did you participate in leisure activities in the last week?

☐ **Never** ☐ **1–2 days** ☐ **3–4 days** ☐ **5–6 days** ☐ **Every day**

3. This is a question about social activity in the last week.

3.1) (Before COVID-19) How many days did you participate in social activities in the last week?

☐ **Never** ☐ **1–2 days** ☐ **3–4 days** ☐ **5–6 days** ☐ **Every day**

3.2) (After COVID-19 onset) How many days did you participate in social activities in the last week?

☐ **Never** ☐ **1–2 days** ☐ **3–4 days** ☐ **5–6 days** ☐ **Every day**

4. This is a question about productive activity in the last week.

4.1) (Before COVID-19) How many days did you participate in productive activities in the last week?

☐ **Never** ☐ **1–2 days** ☐ **3–4 days** ☐ **5–6 days** ☐ **Every day**

4.2) (After COVID-19 onset) How many days did you participate in productive activities in the last week?

☐ **Never** ☐ **1–2 days** ☐ **3–4 days** ☐ **5–6 days** ☐ **Every day**

5. This is a question about <u>education</u> in the last week.

5.1) (Before COVID-19) How many days did you spend engaged in educational activities in the last week?

☐ **Never** ☐ **1–2 days** ☐ **3–4 days** ☐ **5–6 days** ☐ **Every day**

5.2) (After COVID-19 onset) How many days did you participate in education in the last week?

☐ **Never** ☐ **1–2 days** ☐ **3–4 days** ☐ **5–6 days** ☐ **Every day**

III. Nutrition

The following are questions about food intake. Indicate how many times a week the following types of foods have been consumed in the last week.

Please consider your food consumption before and after the onset of the COVID-19 pandemic and mark (V) your answer on the box next to the choice that is most applicable for you.

1. This is a question about the frequency of <u>carbohydrate consumption</u> in the past week.

(※ Carbohydrate-rich food: rice, bread, flour, potato, corn, etc.)

1.1) (Before COVID-19) How often do you consume carbohydrate-rich foods in the last week?

☐ **Never** ☐ **1–2 times per week** ☐ **3–4 times per week** ☐ **5–6 times per week** ☐ **Every day**

1.2) (After COVID-19 onset) How often do you consume carbohydrate-rich foods in the last week?

☐ **Never** ☐ **1–2 times per week** ☐ **3–4 times per week** ☐ **5–6 times per week** ☐ **Every day**

2. This is a question about the frequency of <u>protein consumption</u> in the last week.

(※ Protein-rich food: chicken breasts, fish, tofu, beans, eggs, etc.)

2.1) (Before COVID-19) How often do you consume protein-rich foods in the last week?

☐ **Never** ☐ **1–2 times per week** ☐ **3–4 times per week** ☐ **5–6 times per week** ☐ **Every day**

2.2) (After COVID-19 onset) How often do you consume protein-rich foods in the last week?

☐ **Never** ☐ **1–2 times per week** ☐ **3–4 times per week** ☐ **5–6 times per week** ☐ **Every day**

3. This is a question about the frequency of <u>fat consumption</u> in the last week.

(※ Fatty food: butter, margarine, oil, nuts, etc.)

3.1) (Before COVID-19) How often do you consume fatty foods in the last week?

☐ **Never** ☐ **1–2 times per week** ☐ **3–4 times per week** ☐ **5–6 times per week** ☐ **Every day**

3.2) (After COVID-19 onset) How often do you consume fatty foods in the last week?

☐ **Never** ☐ **1–2 times per week** ☐ **3–4 times per week** ☐ **5–6 times per week** ☐ **Every day**

4. This is a question about the frequency of <u>vitamin consumption</u> in the last week.

(※ Vitamin-rich food: green vegetables, fruit, seaweed, etc.)

4.1) (Before COVID-19) How many vitamin-rich foods did you eat per week?

☐ **Never** ☐ **1–2 times per week** ☐ **3–4 times per week** ☐ **5–6 times per week** ☐ **Every day**

4.2) (After COVID-19 onset) How many vitamin-rich foods you eat per week?

☐ **Never** ☐ **1–2 times per week** ☐ **3–4 times per week** ☐ **5–6 times per week** ☐ **Every day**

5. This is a question about the frequency of <u>mineral consumption</u> in the last week.

(※ Mineral-rich food: dairy products, anchovies, cheese, etc.)

5.1) (Before COVID-19) How often do you consume mineral-rich food in the last week?

☐ **Never** ☐ **1–2 times per week** ☐ **3–4 times per week** ☐ **5–6 times per week** ☐ **Every day**

5.2) (After COVID-19 onset) How often do you consume mineral-rich food in the last week?

☐ **Never** ☐ **1–2 times per week** ☐ **3–4 times per week** ☐ **5–6 times per week** ☐ **Every day**

6. This is a question about the amount of <u>smoking</u> in a week.

6.1) (Before COVID-19) How often did you smoke per week?

☐ **Never** ☐ **1–2 times** ☐ **3–4 times** ☐ **5–6 times** ☐ **Every day**

6.2) (After COVID-19 onset) How often do you smoke per week?

☐ **Never** ☐ **1–2 times** ☐ **3–4 times** ☐ **5–6 times** ☐ **Every day**

7. This is a question about <u>drinking habits</u> in a week.

7.1) (Before COVID-19) How often did you drink alcohol on average per week?

☐ **Never** ☐ **1–2 times** ☐ **3–4 times** ☐ **5–6 times** ☐ **Every day**

7.2) (After COVID-19 onset) How often do you drink alcohol on average per week?

☐ **Never** ☐ **1–2 times** ☐ **3–4 times** ☐ **5–6 times** ☐ **Every day**
